# Death of Prof. Blandin

**Published:** 1849-08

**Authors:** 


### DEATH OF PROFESSOR BLANDIN.

By one of the late transatlantic steamers, intelligence has been received of the death of P. Frederick Blandin, M. D. Dr. Blandin, at the time of his death, occupied the chair of Operarive Surgery in the School of Medicine, at Paris, and was one of the lecturers on clinical surgery at the Hôtel Dieu Hospital. He died after a brief illness, with cholera, aged 50 years. There were few of the surgeons of the French capital better known in this country than Dr. Blandin. An estimable gentleman, an ornament to society, an able writer, a brilliant operator, and a successful teacher. His loss will be everywhere deeply felt by the members of the profession of which he was one of the brightest ornaments.

